# Molecular Dynamics Simulation on Cutting Mechanism in the Hybrid Machining Process of Single-Crystal Silicon

**DOI:** 10.1186/s11671-021-03526-x

**Published:** 2021-04-21

**Authors:** Changlin Liu, Wenbin He, Jianning Chu, Jianguo Zhang, Xiao Chen, Junfeng Xiao, Jianfeng Xu

**Affiliations:** State Key Laboratory of Digital Manufacturing Equipment and Technology, School of Mechanical Science and Engineering, Huazhong University of Science and Technology, Wuhan, 430074 China

**Keywords:** Molecular dynamics simulation, Single-crystal silicon, Hybrid machining process, Cutting mechanism

## Abstract

In this paper, molecular dynamics simulations are carried out to investigate the cutting mechanism during the hybrid machining process combined the thermal and vibration assistants. A modified cutting model is applied to study the material removal behavior and subsurface damage formation in one vibration cycle. The results indicate that during the hybrid machining process, the dominant material removal mechanism could transform from extrusion to shearing in a single vibration cycle. With an increase of the cutting temperature, the generation and propagation of cracks are effectively suppressed while the swelling appears when the dominant material removal mechanism becomes shearing. The formation mechanism of the subsurface damage in one vibration cycle can be distinct according to the stress distribution. Moreover, the generation of the vacancies in workpiece becomes apparent with increasing temperature, which is an important phenomenon in hybrid machining process.

## Introduction

Single-crystal silicon is an important semiconductor material, which has been widely used in infrared optics, microelectronics, and optoelectronics systems owing to its excellent optical and mechanical properties [1, 2]. However, due to the hardness and brittleness nature of single-crystal silicon, microscopic brittle fracture and subsurface damage can be generated during mechanical machining. During micro-milling process, machining-induced interior type edge chipping defects can be generated in workpiece [3]. In single-crystal diamond cutting (SPDT) machining, a damaged layer ranges from 200 to 600 nm can be formed depending on processing parameters [4, 5]. Although the subsurface damage layer can be decreased to about 50 nm by grinding and polishing. The machining efficiency and ability in fabricating complex structures are limited. To overcome these problems, various assistive machining technologies have been proposed and tested. In particular, the thermal assisted cutting (TAC) [6] and vibration assisted cutting (VAC) [7] have attracted widespread attention as its extraordinary cutting performance.

For brittle materials like single-crystal silicon, the brittle-to-ductile transition can be promoted when the machining temperature is increased. During TAC process, the silicon workpiece is thermally softened, which causes the decrease of the cutting forces [8] and specific cutting energy [9, 10]. Meanwhile, the annealing of the high-pressure phases into cubic silicon phase become apparent when the machining temperature is increased [11]. With appropriate selection of the machining parameters, desired machined surface with high phase purity and low subsurface damage can be achieved by TAC [12–14]. In addition to TAC, the vibration assisted cutting (VAC) is another promoting method to achieve high-quality surface on single-crystal silicon. This technique has been applied in the manufacturing industry since 1960s [15]. In the early development of this technology, only a linear vibration motion in the nominal cutting direction is practiced in machining, which is named as linear vibration cutting (LVC). In 1994, the elliptical vibration cutting (EVC) was proposed by Shamoto and Moriwaki [16]. As follows, the machining feasibilities of EVC on many brittle materials like silicon [17, 18], reaction-bonded silicon carbide [7], tungsten carbide [19, 20], and harden steel [21] has been verified. During EVC process, the subsurface damage can be effectively suppressed since the transient depth of cut (DOC) is much smaller than the nominal DOC [22]. Besides, because of the separation in each vibration cycle, the contact surfaces between the cutting tool and workpiece are exposed into the surrounding gas or fluid, which dissipates the generated cutting heat. Therefore, cutting tool wear like adhesion and thermo-chemical reaction [23] can be effectively suppressed.

To further improve the machinability of brittle materials, hybrid machining (HM) experiments of combining the thermal and vibration assistant have been conducted [24, 25]. It was found that when cutting Inconel 718 by HM method, the machined surface roughness can be effectively decreased [26]. Through experiments and finite element method (FEM) simulations, a substantial drop of the cutting forces and a superior surface quality of titanium alloys can be achieved during HM process [27]. These results demonstrate the feasibility of HM method in precision machining of brittle materials. However, it is hard to directly observe and measure the physical variables during machining process since the cutting tool vibrates in a high frequency and the deformation zone is at high temperature. Furthermore, in nanometric surface fabricating, the transient material removal thickness usually ranges from sub-nanometers to a few nanometers. Therefore, the traditional continuum representation of the problem such as FEM is questionable since the quantum mechanical effects becomes apparent.

In recent years, molecular dynamics (MD) simulation has been widely applied in investigations of the assistive machining process due to its advantages in studying nanometric cutting process [28–30]. Based on previous simulations of TAC [31], when the cutting temperature is increased, the anisotropy in the cutting force, specific cutting energy, and yielding stress becomes more obvious. Meanwhile, the shear force in workpiece is lower at higher cutting temperature, which leads to narrower shear zones and higher magnitudes of the shear plane angle [32]. Furthermore, the material removal rate can be improved with increasing cutting temperature since more chips is formed [33]. For EVC process, it has been discovered by MD simulation that the compressive stress and shear stress in the deformation region can be greatly decreased compared with ordinary cutting [34], which is advantageous for subsurface damage suppression. Besides, EVC process shows obvious thinning of cutting chips, resulting in an increase in the ratio of uncut chip thickness to cut chip thickness [35]. Furthermore, it has been unraveled that the vibration parameters including amplitudes ratios, vibration frequencies, and phase differences have great influences on the material removal performance [34, 36].

These remarkable achievements have improved the understanding of the machining mechanism for assistive machining process. However, in order to save the computation time and memory, the simulation systems are usually quite small. In previous simulations of EVC process, the vibration amplitudes and the nominal DOC are less than 5 nm [22, 36]. Therefore, the transient material removal thickness is usually less than 1 nm, which fails to describe the actual material removal process accurately. Moreover, the MD simulations of the HM process has not been reported. The mechanism of the material removal process and subsurface damage formation during HM process is still unclear. Therefore, in this paper, MD simulation is carried out to reveal the cutting mechanism of HM process. The classic cutting model is modified that the vibration parameters are much closer to the experimental values, e.g., the vibration amplitude is enlarged to 40 nm with a nominal cutting speed of 3.125 m/s. The material removal mechanism in one vibration cycle and the influence of increased cutting temperature are investigated. MD simulation is conducted by the famous Large-scale Atomic/Molecular Massively Parallel Simulator (LAMMPS) [37]. The post processing software OVITO [38] is employed to analyze the simulation results.

## Simulation Method

### Details of the Cutting Model

**Fig. 1:**
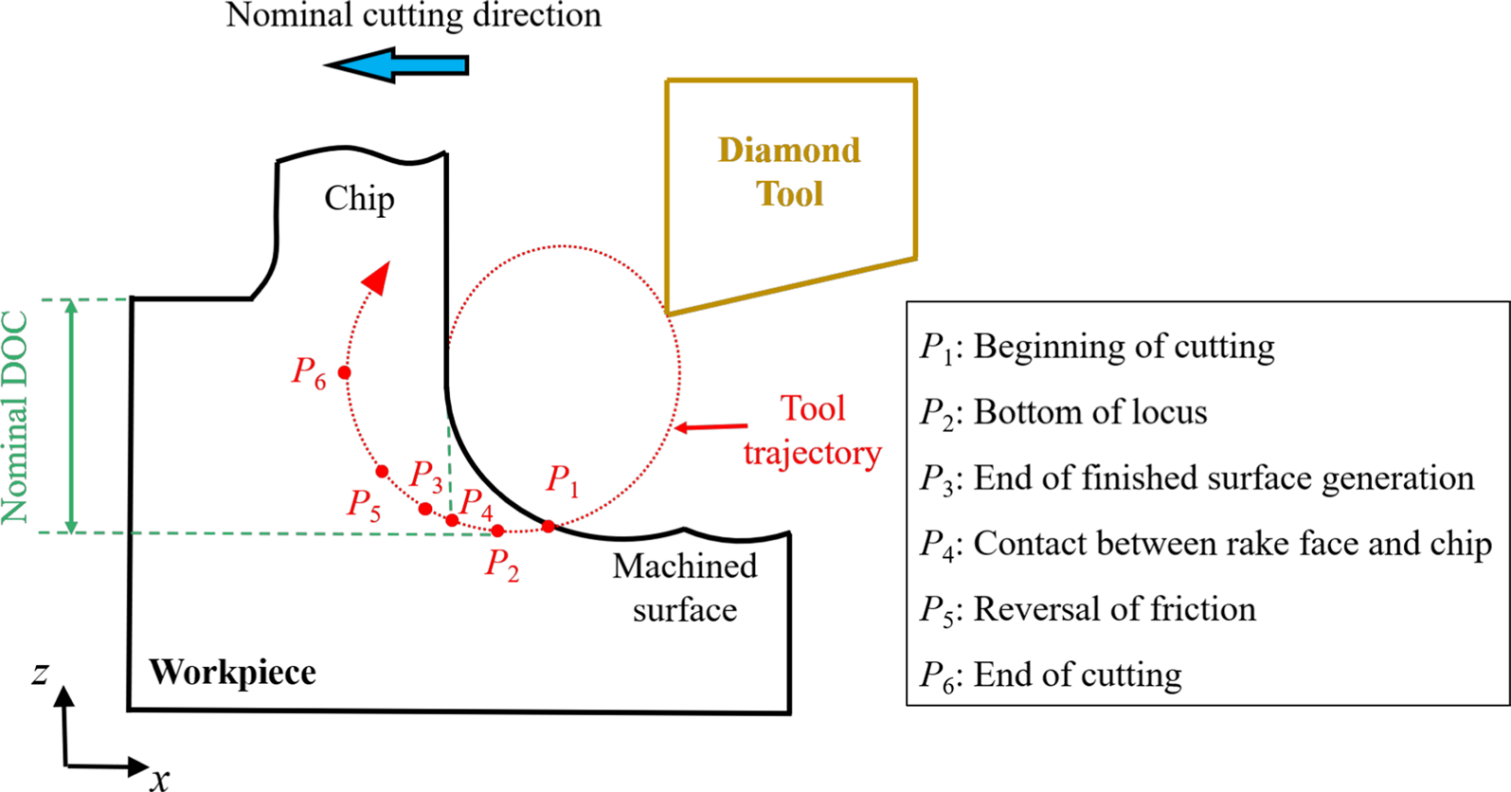
The schematic diagram of the EVC process

Figure 1 shows the schematic diagram of the EVC process, which is originally presented by Shamoto et al. [39]. The tool trajectory can be expressed as:

1 $$x\left( t \right) = A_{{\text{c}}} \sin \left( {2\pi ft} \right) - vt$$

2 $$z\left( t \right) = A_{{\text{d}}} \sin \left( {2\pi ft + \varphi } \right)$$

where *x*(*t*) and *z* (*t*) represent the cutting tool displacement in the *x* and *z* directions. *A*_c_ and *A*_d_ are the vibration amplitude in the nominal cutting direction (*x* direction) and the nominal DOC direction (negative *z* direction). Parameters *f*, *v*, *φ*, and *t* represent the vibration frequency, nominal cutting speed, phase difference, and simulation time, respectively. The simulation time *t*_*i*_ represents the time of the point *P*_*i*_ on the tool trajectory from Fig. 1.

According to the geometrical relationship [40], the value of *t*_1_ and *t*_3_ can be determined by:

3 $$x\left( {t_{1} } \right){-}x\left( {t_{3} } \right) = 2\pi v/\omega$$

4 $$z\left( {t_{1} } \right){-}z\left( {t_{3} } \right) = 0$$

Then, *t*_6_ can be obtained when the transient movement direction of the diamond tool is parallel to tool rake face:

5 $$\frac{{A_{{\text{c}}} \sin (2\pi ft_{6} ) + v}}{{A_{{\text{d}}} \sin (2\pi ft_{6} + \varphi ) }} = \tan \gamma$$

where *γ* is the rake angle of diamond cutting tool.

**Fig. 2:**
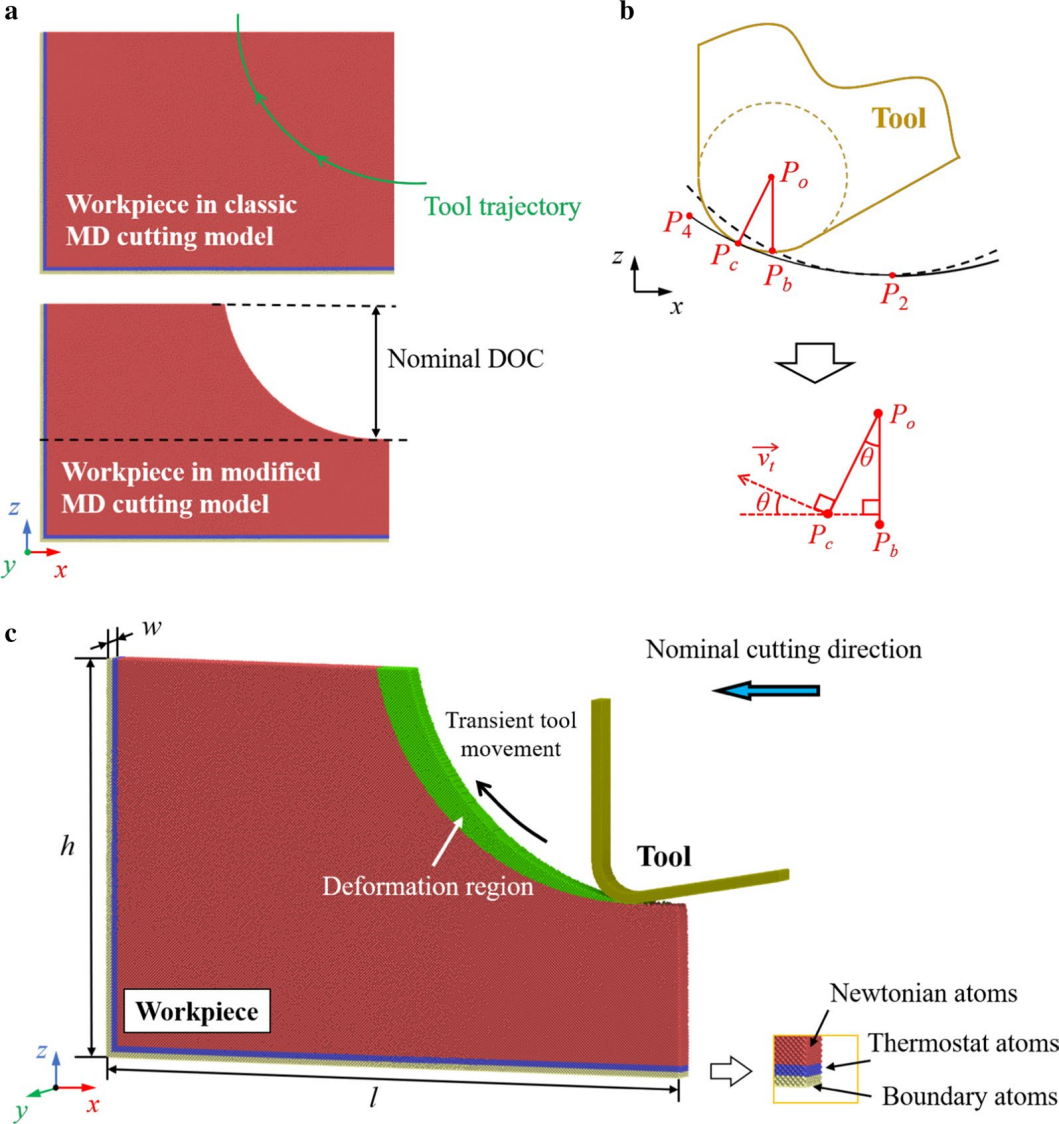
Scheme of the MD cutting model. **a** Modification of the workpiece. **b** Determination of the tool trajectory. **c** The morphology of the modified MD model. Green atoms represent the deformation region in one vibration cycle

The MD model is present in Fig. 2. The single-crystal silicon workpiece is set as deformable body. While the diamond tool is regarded as rigid body since the tool wear can be neglected in this simulation. The morphology of workpiece in the classic cutting model is reshaped according to tool trajectory in the previous vibration cycle with consideration of the tool edge radius. The tool trajectory can be determined as illustrated in Fig. 2b. *P*_o_ and *P*_b_ are the center and the bottom point of the tool edge circle. When the tool edge effect is considered, the transient surface generation point *P*_c_ varies along the tool edge during the tool movement. The actual finished surface is generated by the envelope line of the cutting tool edge. If the trajectory of *P*_b_ is expressed by Eqs. (1) and (2), the trajectory of *P*_c_ can be calculated through [41]:

6 $$x_{{\text{c}}} \left( t \right) = A_{{\text{c}}} \sin \left( {2\pi ft} \right) - vt - r\sin \theta \left( t \right)$$

7 $$z_{{\text{c}}} \left( t \right) = A_{{\text{d}}} \sin \left( {2\pi ft + \varphi } \right) + r(1{-}\cos \theta \left( t \right))$$

where

8 $$\sin \theta \left( t \right) = \frac{{z^{{\prime }} (t)}}{{\sqrt {x^{{\prime }} (t)^{2} + z^{{\prime }} (t)^{2} } }}$$

9 $$\cos \theta \left( t \right) = \frac{{ - x^{{\prime }} (t)}}{{\sqrt {x^{{\prime }} (t)^{2} + z^{{\prime }} (t)^{2} } }}$$

Silicon atoms are divided into three groups: boundary atoms, thermostat atoms, and Newtonian atoms. The boundary atoms are fixed in their balanced positions to hold the workpiece during simulation. The thermostat atoms are kept at the ambient temperature to dissipate the generated cutting heat while Newtonian atoms follow the Newton’s second law.

Details of the simulation parameters are listed in Table 1. The length *l* and height *h* were determined to keep enough distance between the cutting zone and the fixed boundaries. The periodic boundary condition is applied along the *y* direction to mimic bulk silicon. The nominal cutting direction, tool rake/clearance angle, and phase difference were determined referring to the experimental setup [42]. The vibration amplitude and nominal DOC are enlarged to approach the experimental scale with acceptable simulation cost. Meanwhile, in order to ensure the thickness of removed material (green atoms in Fig. 2c), the speed ratio and the vibration frequency were set as 40 and 500 MHz, respectively. Therefore, the nominal cutting speed was determined as 3.125 m/s. Furthermore, simulations with different cutting temperature are conducted to reveal the effect of thermal assistant on cutting mechanism. The cutting temperature is increased from 300 to 1200 K, which is realizable during TAC like laser assisted machining [4, 11].

**Table 1:** Parameters of the MD simulation model

Parameters	Value
Length *l*	108.6 nm
Height *h*	70.6 nm
Width *w*	4.3 nm
Nominal cutting direction	(001)/[$\stackrel{\mathrm{-}}{1}$00]
Tool rake/clearance angle	0°/10°
Total atoms in workpiece	1,327,001
Phase difference *φ*	90°
Amplitude in the nominal cutting direction *A*_c_	40 nm
Amplitude in the DOC direction *A*_d_	40 nm
Nominal DOC	40 nm
Vibration frequency *f*	500 MHz
Nominal cutting speed	3.125 m/s
Tool edge radius	7.14 nm
Ambient temperature	300 K, 600 K, 900 K, 1200 K
Timestep	1 fs

In this modified model, only the cutting stage during the vibration cycle is simulated and the timesteps when the workpiece is separated with cutting tool are saved. Therefore, the computation power can be concentrated on the transient cutting process. Most importantly, the transient material removal process can be described accurately. A comparison between the modified model and classic MD model is shown in Table 2.

**Table 2:** Comparison between the parameters between classic model and modified model

Parameters	Experiments [42, 43]	Classic model [22, 36, 44]	Modified model
Vibration amplitude	> 500 nm	< 5 nm	40 nm
Nominal DOC	~ 100 nm	< 5 nm	40 nm
Nominal cutting speed	~ 0.01 m/s	> 50 m/s	3.125 m/s
Vibration frequency	~ 50 kHz	> 10 GHz	500 MHz

### Potential Function

In MD simulation, it is important to adopt a robust potential to describe the interaction between atoms. For single-crystal silicon, scholars have developed many potentials such as the modified embedded-atom method (MEAM) [45], Stillinger–Weber (SW) [46], Tersoff [47] and charge optimized many-body (COMB) [48] potentials. Among these potentials, the analytical bond-order potential (ABOP) proposed by Erhart and Albe [49] has attracted growing attention. It is a three-body potential function which allows formation and breaking of bonds during the machining simulation. According to previous researches [50], the ABOP can describe both dimer and bulk properties of silicon accurately. Meanwhile, the mechanical properties of silicon made by the ABOP are consistent with the experiments well [31], which is important in MD simulations of nanoscale machining. Therefore, in this paper, the ABOP potential is applied to describe the silicon–silicon and carbon–carbon interactions. Meanwhile, the interaction of silicon-carbon is described by the Morse potential, which has been proved as an efficient potential in nanoscale cutting simulation [51, 52]. The Morse potential function can be expressed as:

10 $$E_{{\text{Si - C}}} \left( {r_{ij} } \right) \, = D_{{\text{M}}} \left[ {{\text{e}}^{{ - 2a(r_{ij} - R_{{\text{M}}} )}} - 2{\text{e}}^{{ - a(r_{ij} - R_{{\text{M}}} )}} } \right]$$

where *D*_M_*, a,* and *R*_M_ represents the cohesion energy, modulus of elasticity, and the equilibrium distance between atoms, respectively. The parameters for Morse potential is [53]: *D*_M_ = 0.435 eV, *a* = 46.487 nm^−1^, *R*_M_ = 0.19475 nm.

## Results and Discussion

### Cutting Performance

**Fig. 3:**
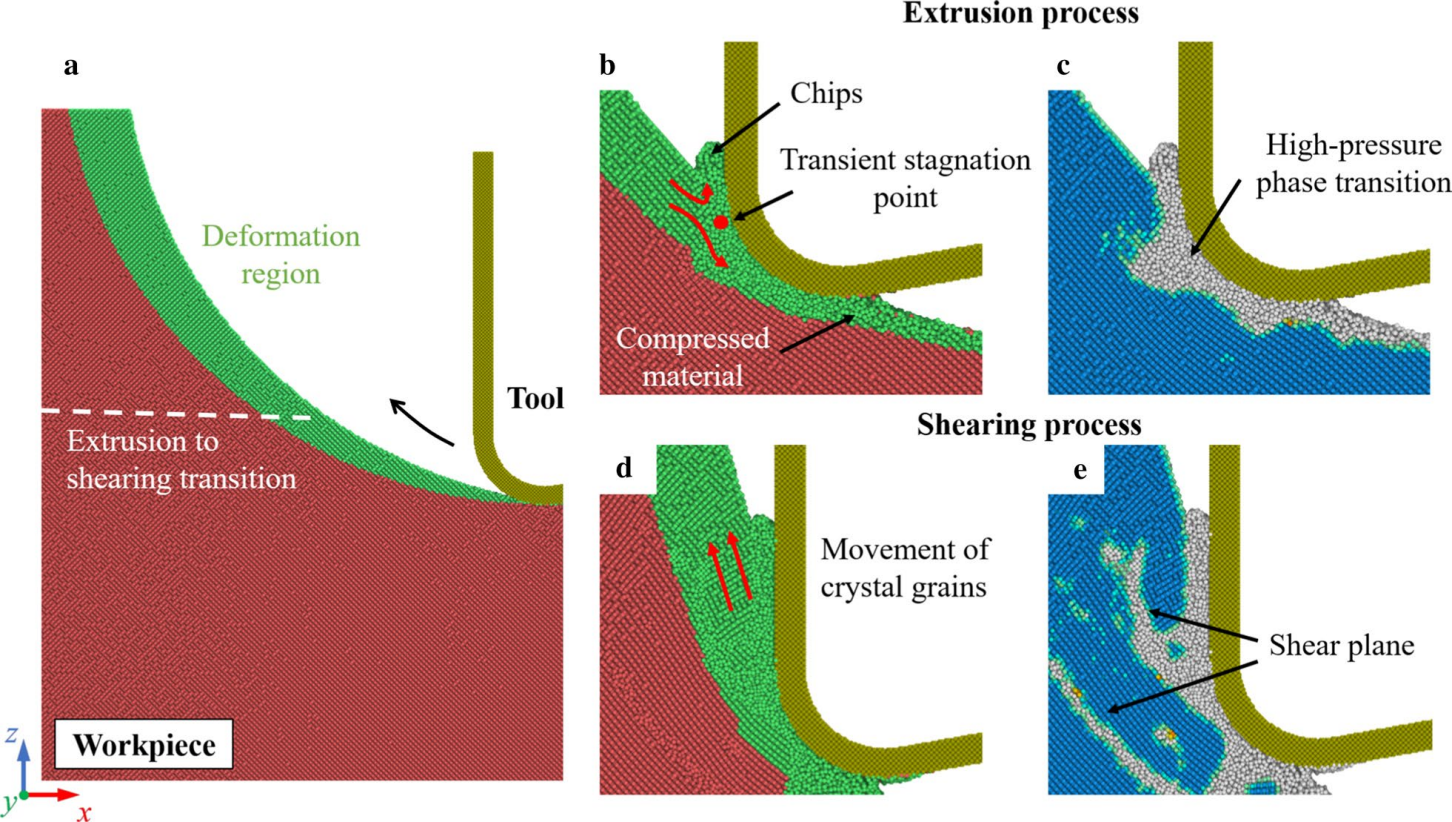
Transition of the material removal mechanism. **a** Illustration of the deformation region. **b**, **d** Extrusion and shearing process. **c**, **e** Identification of the crystal structure in workpiece. Blue atoms represent the cubic diamond structure while the gray atoms are in the non-diamond structure

In ordinary cutting, the dominant material removal mechanism can be greatly influenced by the undeformed chip thickness [54]. With small undeformed chip thickness, the dominant material removal mechanism is extrusion. The metallic stable phase (Si-II) can be generated by the high-pressure phase transition (HPPT), which facilities the ductile deformation of silicon. When the undeformed chip thickness is increased, material can be mainly removed via shearing process. While in EVC process, since the undeformed chip thickness is constantly varying, the material removal mechanism can transform from extrusion to shearing in one vibration cycle. Figure 3 shows the snapshots of the cutting simulation at 300 K. The crystal structure of workpiece is determined by the common neighbor analysis (CNA) [55]. This analysis finds atoms that are arranged in a cubic or hexagonal diamond lattice. The non-diamond structure in Fig. 3c, e mainly contains the amorphous phase (a-Si), Si-II, and other defective atoms [56]. These structures are unstable and will transform into a-Si after cutting. It can be observed from Fig. 3b, c that material is mainly removed through extrusion in the initial cutting stage. A transient stagnation point can be observed near the cutting tool edge. Similar with ordinary cutting, materials in the deformation region is divided by the stagnation point into chips and compressed material. As the cutting tool proceeding, the undeformed chip thickness is increased. Shear planes and polycrystal grains are generated in the workpiece, indicating that shearing becomes the dominant material removal mechanism.

**Fig. 4:**
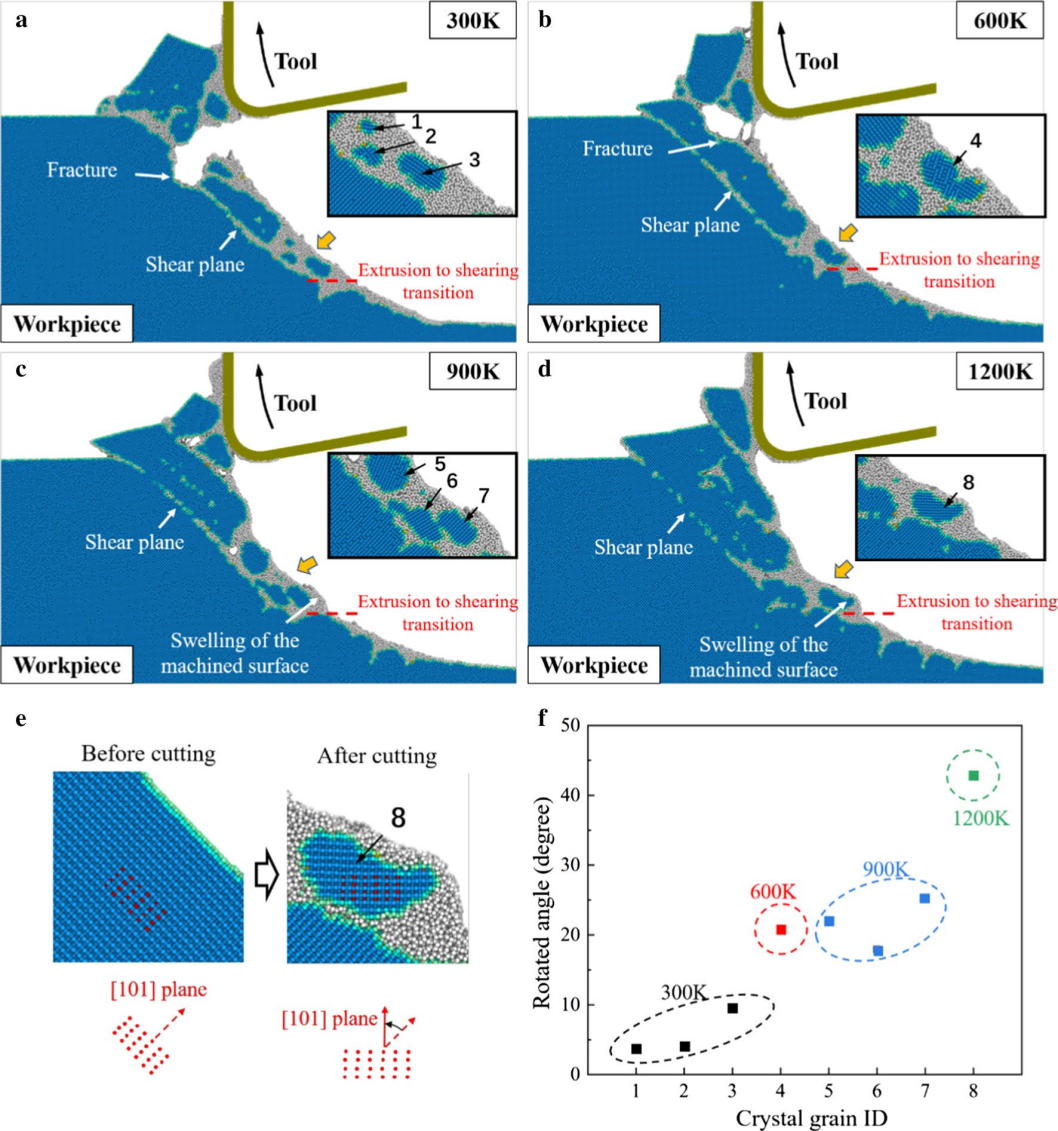
Workpiece morphology of the HM process at **a** 300 K. **b** 600 K. **c** 900 K. **d** 1200 K. Blue atoms represent the cubic diamond structure while the gray atoms are in non-diamond structure. **e** Determination of the rotation angle of crystal grains. **f** The rotation angle with increasing cutting temperature

Figure 4 shows the workpiece morphology at different cutting temperatures. At 300 K, obvious crack and fracture can be observed in workpiece during the tool upward movement. For brittle material like single-crystal silicon, the tool upward movement would lead to tearing off of materials and leave defects in the workpiece, which is considered as a specific problem in EVC [42]. Although these cracks can be removed by further vibration cycles, the machining stability will be impacted due to the irregularity of workpiece surface. When the cutting temperature is increased, the generation and propagation of cracks is effectively suppressed. From Fig. 4d, no obvious fracture is detected when the cutting temperature raises to 1200 K. However, it is observed that at 900 K and 1200 K, swelling of the machined surface becomes obvious when the material removal mechanism transformed into shearing. It can be concluded that as more crystal grains are generated in shearing stage, the swelling can be caused by the rotation of these crystal grains at high temperature.

**Fig. 5:**
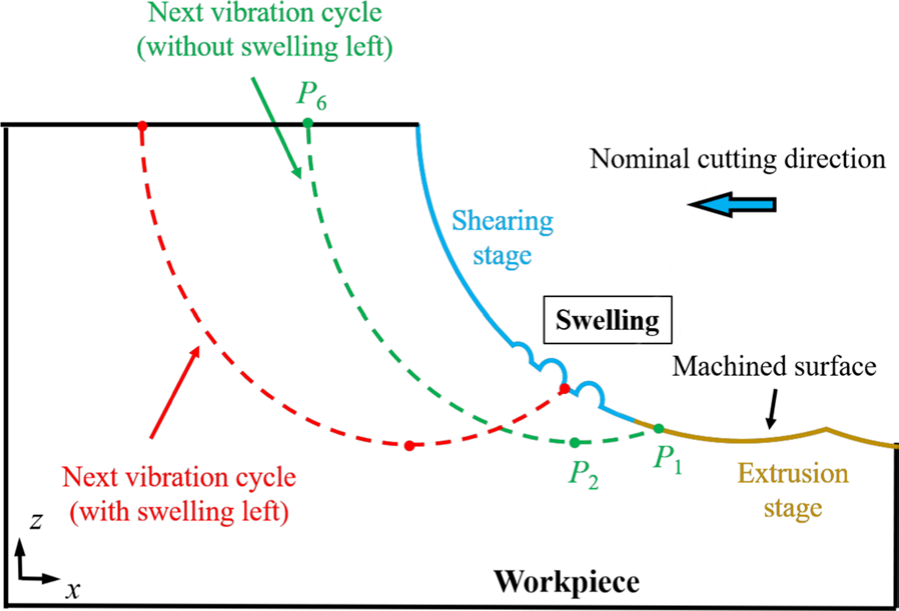
Elimination of swelling in HM process

For a clear description of this rotation, the coordinates of 24 marked atoms (red atoms) in the crystal grains are used to calculate the average rotation angle, as illustrated in Fig. 4e. The rotation angle of 8 crystal grains (numbered in Fig. 4a–d) is summarized in Fig. 4f. It can be observed that the rotation angle is obviously increased at elevated temperature. During the HM process, the viscosity of a-Si can be greatly decreased at high temperature and the pulling-up motion of workpiece atoms are promoted by the tool upward movement. Therefore, the atomic flow in workpiece is enhanced and the rotation of the crystal grains can be facilitated, leading to swelling of the machined surface. To restrain the rotation of crystal grains, the heating power should be controlled to avoid overheating of the workpiece. Besides, the vibration parameters should be chosen carefully, e.g., smaller nominal cutting speed and higher vibration frequency should be applied to suppress the generation of crystal grains and remove the swelling by further vibration cycles. As illustrated in Fig. 5, with appropriate vibration parameters, *P*_1_ could locate in the extrusion stage and the final machined surface is generated through extrusion without swelling.

### Stress Field in Workpiece

To further investigate the cutting mechanism during HM process, the stress distribution in workpiece was calculated. In MD simulation, the hydrostatic stress can be expressed as:

11 $$\sigma_{{{\text{hydrostatic}}}} = \, (\sigma_{x} + \sigma_{y} + \sigma_{z} )/3$$

where *σ*_*x*_, *σ*_*y*_, and *σ*_*z*_, are stress tensors from LAMMPS output data.

**Fig. 6:**
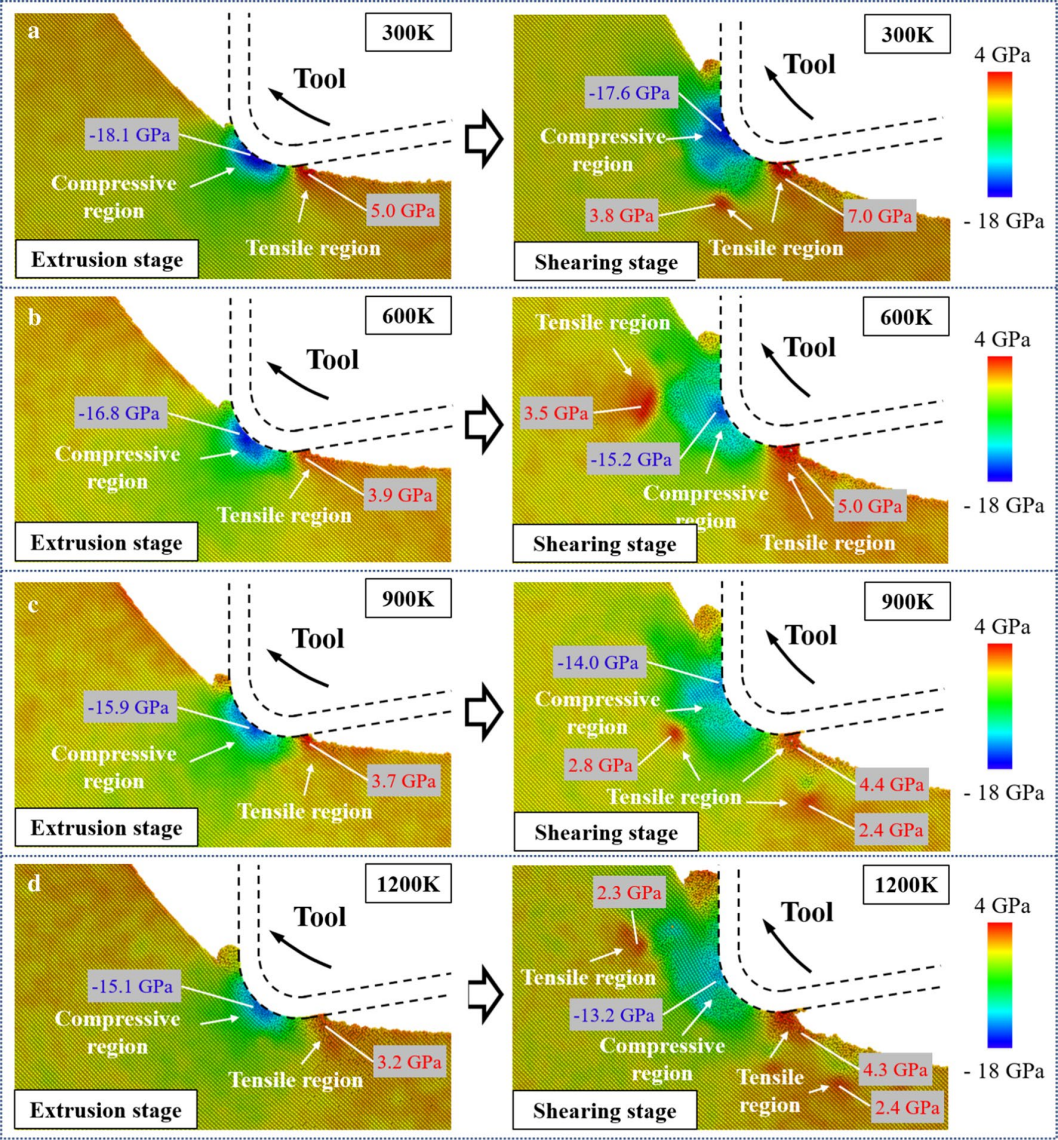
The hydrostatic stress distribution at: **a** 300 K. **b** 600 K. **c** 900 K. **d** 1200 K

The hydrostatic stress distribution during extrusion and shearing stages is shown in Fig. 6. And the peak values of the stresses in the compressive and tensile regions were marked. With the tool movement, the contact point between tool and workpiece varies along the tool edge cycle, which results in the movement of the compressive region from the tool edge to the rake face. Following previous reports, the HPPT from single-crystal silicon phase (Si-I) to Si-II could occur at pressures starting at 10–12 GPa [57, 58]. In the cutting simulation at 300 K, the max compressive stress in extrusion and shearing stage reached to 18.1 GPa and 17.6 GPa, respectively. This result indicates that the ductile Si-II phase can be generated during cutting and the HPPT still exists in the shearing stage. In addition, in the extrusion stage, the tensile stress mainly concentrates near the contact area between the tool flank face and machined surface as the result of the adhesion of silicon atoms and tool surface. As tool proceeds into shearing stage, the tensile region is enlarged and the tensile stress concentration in the subsurface workpiece is greatly increased, which is caused by the pulling-up motion. When the cutting temperature is increased, the plastic deformability of single-crystal silicon is improved and internal stress in the workpiece is decreased. As the temperature increases from 300 to 1200 K, the max compressive stress decreased 16.6% and 25% in extrusion and shearing stage. Meanwhile, although the tensile stress concentration in the subsurface workpiece is still obvious, the peak value of the tensile stress is apparently decreased for more than 30%. It has been reported that the fracture toughness of single-crystal silicon can be effectively increased at higher temperature [59]. Therefore, cracks and fractures caused by the torn-off effect due to tool upward movement can be effectively suppressed.

**Fig. 7:**
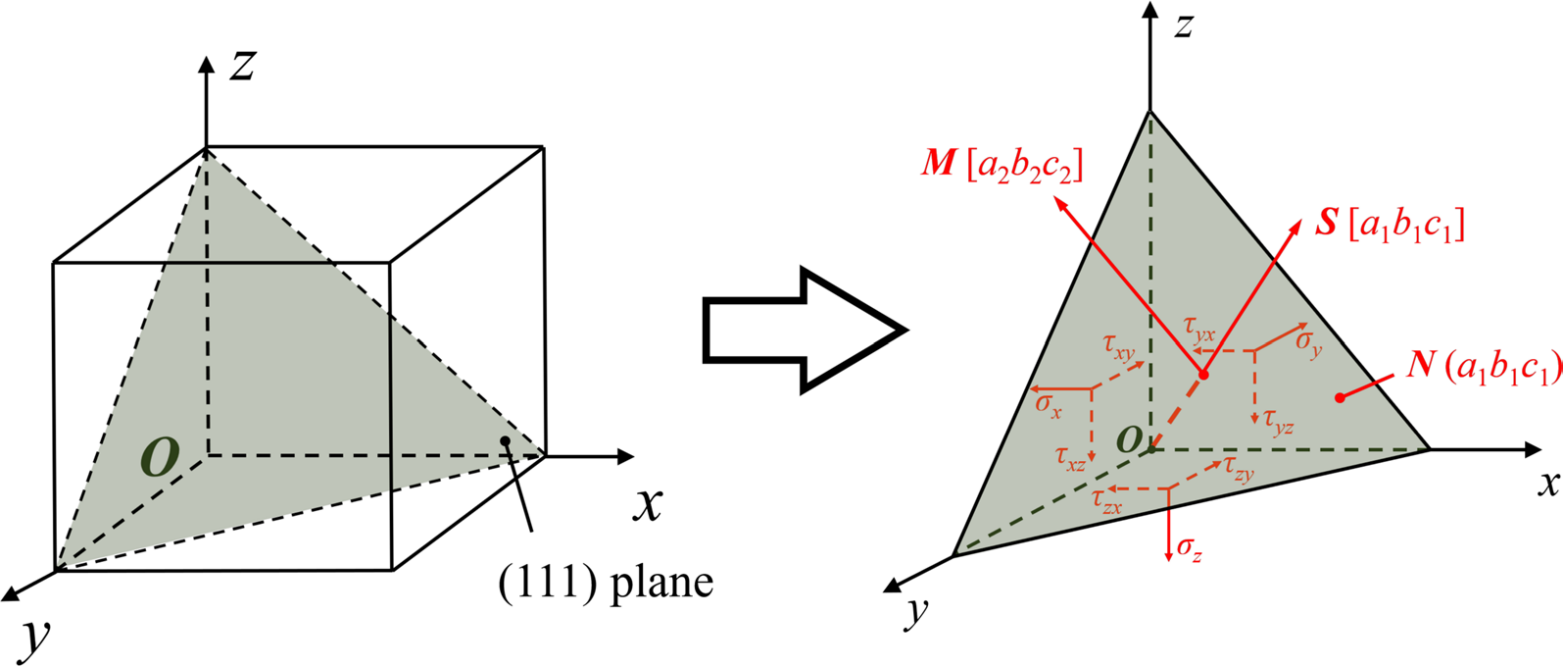
Illustration of the stress tensors

Single-crystal silicon has a Face Center Cubic (FCC) crystal structure with 12 slip systems. Based on the tool movement, the main slip systems for shear deformation are the (111)/[$\stackrel{\mathrm{-}}{1}$01] and (1$\stackrel{\mathrm{-}}{1}$1)/[$\stackrel{\mathrm{-}}{1}$01] systems. Therefore, the resolved shear stress component *τ*_*s*_ in the (111)/[$\stackrel{\mathrm{-}}{1}$01] slip system is calculated. As illustrated in Fig. 7, the resolved shear stress component *τ*_s_ in the direction ***M*** of the slip plane ***N*** can be calculated through stress tensors by:

12 $$\tau_{{\text{s}}} = a_{1} a_{2} \sigma_{x} + b_{1} b_{2} \sigma_{y} + c_{1} c_{2} \sigma_{z} + \left( {a_{1} b_{2} + a_{2} b_{1} } \right)\tau_{xy} + \left( {a_{1} c_{2} + a_{2} c_{1} } \right)\tau_{xz} + \left( {b_{1} c_{2} + b_{2} c_{1} } \right)\tau_{yz}$$

where *a*_1_, *b*_1_, *c*_1_ are the direction cosines of the normal direction of plane ***N*** while *a*_2_, *b*_2_, *c*_2_ are the direction cosines of the slip direction ***M***. While *τ*_*xy*_, *τ*_*xz*_, and *τ*_*yz*_ are the shear stress tensors from LAMMPS output data.

**Fig. 8:**
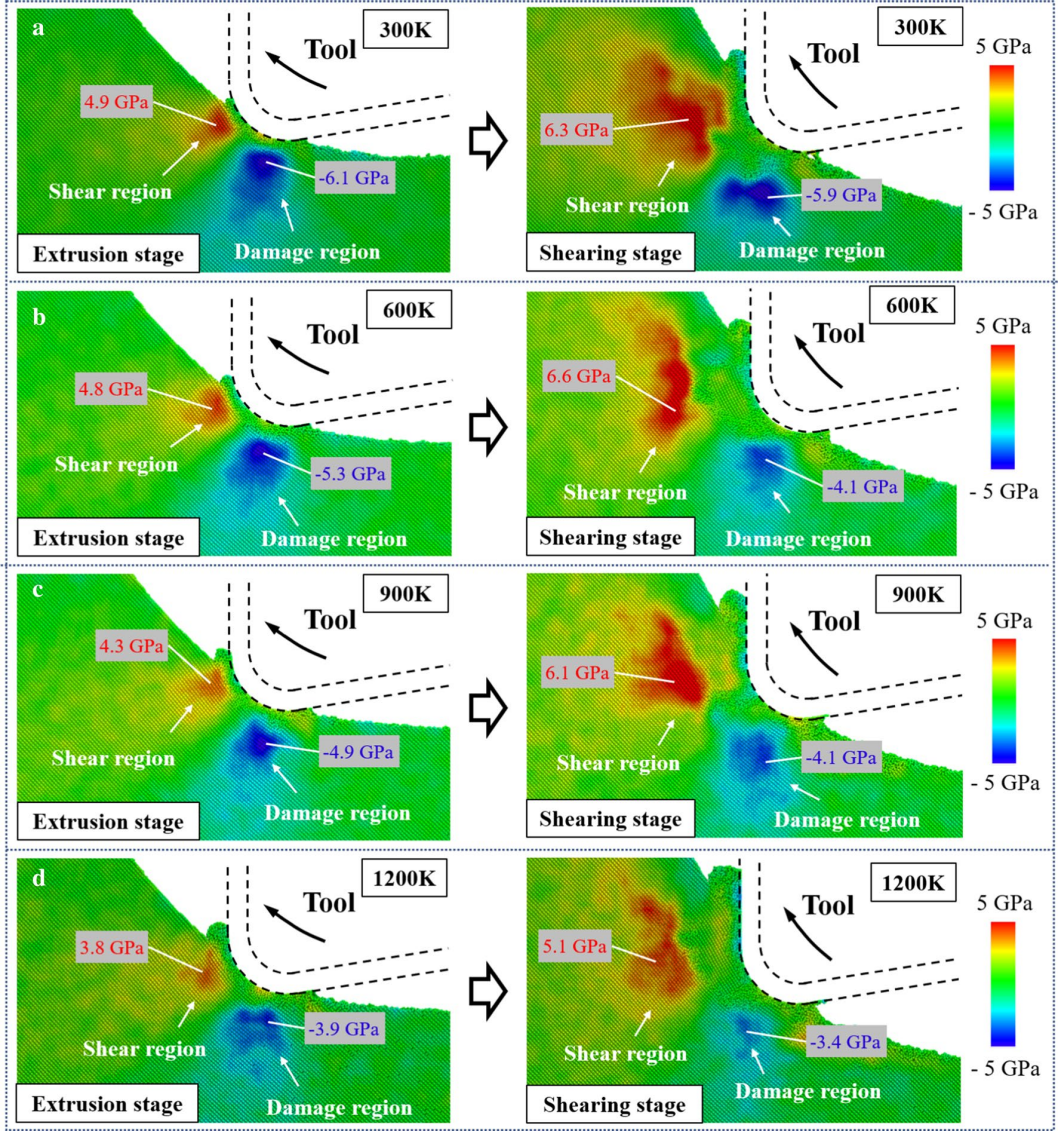
The resolved shear stress distribution at: **a** 300 K. **b** 600 K. **c** 900 K. **d** 1200 K

The distribution of the resolved shear stress *τ*_*s*_ is shown in Fig. 8. The region with positive *τ*_s_ is defined as the shear region since the slip motion along the [$\stackrel{\mathrm{-}}{1}$01] direction is promoted, which facilitates the material removal through shearing. While the region with negative *τ*_s_ is regarded as the damage region because the slip motion is preferable in the oppose direction, leading to the formation of subsurface damage in workpiece. In the extrusion stage, the stress in the shear region is smaller than that in the damage region. Subsurface damage caused by shear deformation can be generated beneath the machined surface [60]. As the cutting tool movement, the shear stress along the [$\stackrel{\mathrm{-}}{1}$01] direction is gradually increased, causing the material removal transition from extrusion to shearing. Besides, since the position of the damage region moves upward along the tool movement, the generated damage can be removed through further vibration cycle and will not left in workpiece. When the temperature is increased from 300 K to 1200 K, the shear stress in the damage region decreased 36.1% and 42.4% in the extrusion and shearing stage, respectively. In contrast, due to the tool upward motion, the decrease of the shear stress along the [$\stackrel{\mathrm{-}}{1}$01] direction in shearing stage is much less apparent. The critical resolved shear stress (CRSS) for slip motion can be expressed as [61]:

13 $$\tau_{{\text{c}}} \left( T \right) = C\varepsilon^{1/n} \exp \left( \frac{U}{nkT} \right)$$

where *U* and ε represent the activation energy of glide movement and the strain rate. Parameters *n* and *C* are material constants. It can be concluded that the CRSS can be decreased obviously with increasing temperature. Therefore, the shear deformation in the [${\overline{\text{1}}}$01] direction can be facilitated at elevated temperature.

### Phase Transition

**Fig. 9:**
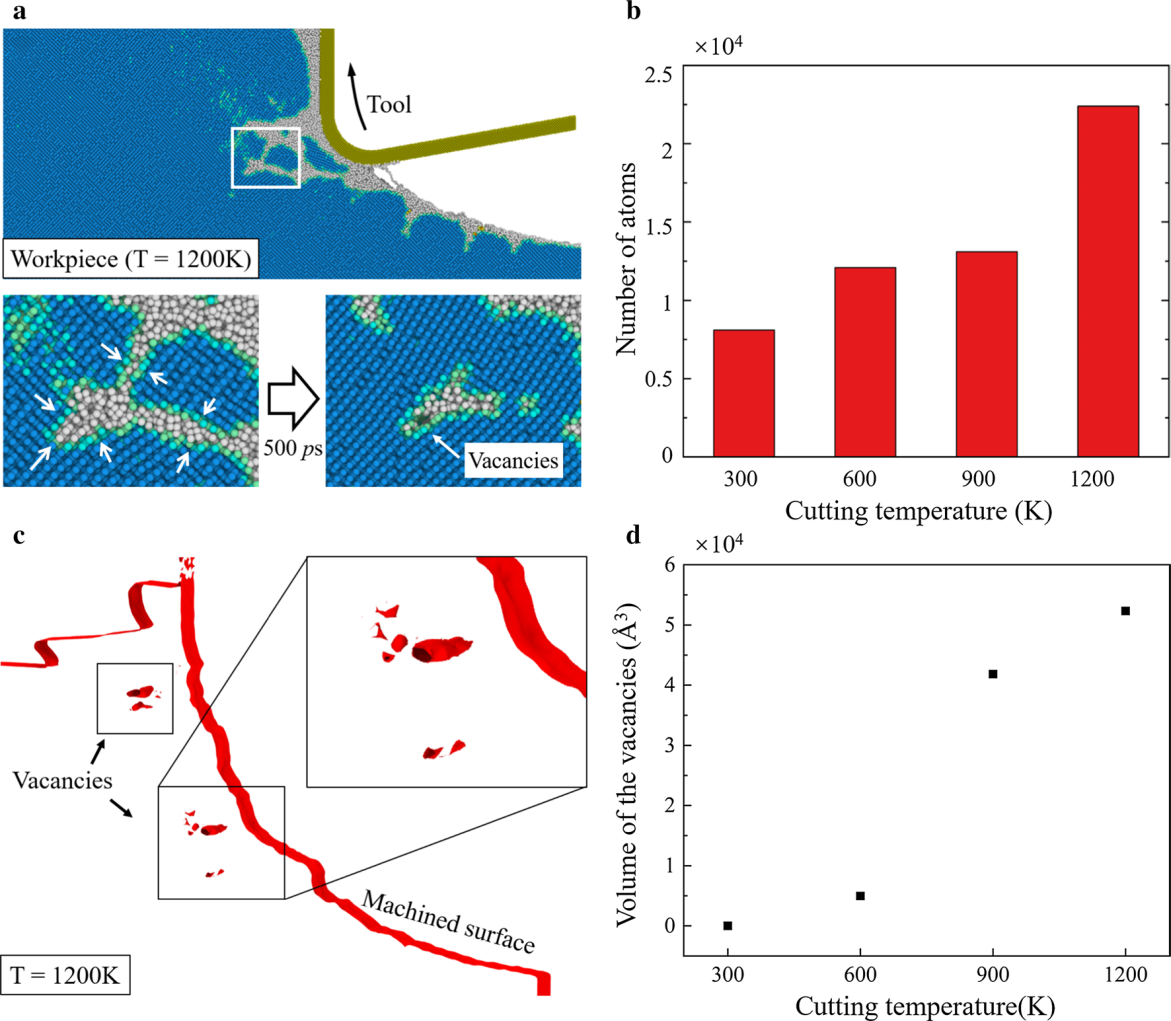
Relaxation process and vacancies in workpiece. **a** Snapshot of the damage pattern of workpiece at 1200 K. **b** Number of the atoms transformed from the non-diamond structure into Si-I phase. **c** Constructed surface mesh of the workpiece at 1200 K. **d** The volume of the vacancies at different temperatures

When the cutting temperature is increased, the phase transition of silicon can be greatly influenced. The relaxation of the a-Si and transition to Si-I can be promoted at an appropriate temperature [62]. In Fig. 4, the damage pattern in the workpieces becomes narrower at high temperature. A detailed observation of the damage pattern when cutting at 1200 K is present in Fig. 9a. It is observed that the generated damage in the deformation region is partly recovered after cutting, indicating that the transition from the non-diamond structure to Si-I has occurred. And more Si-I atoms are generated when the cutting temperature is increase, as shown in Fig. 9b. Furthermore, the constructed surface mesh (red color) [63] of the machined workpiece at 1200 K is present in Fig. 9c. It is observed that some vacancies are formed in the subsurface workpiece. Since the atoms are more tightly packed in Si-I phase, the transition to Si-I could cause a shrink of the material, which induces vacancies in workpiece. The volume of the vacancies at different temperatures is calculated and present in Fig. 9d. It is observed that nearly no vacancies are generated at room temperature. While obvious increase of the vacancies can be detected when the cutting temperature is increases to 900 K and 1200 K.

**Fig. 10:**
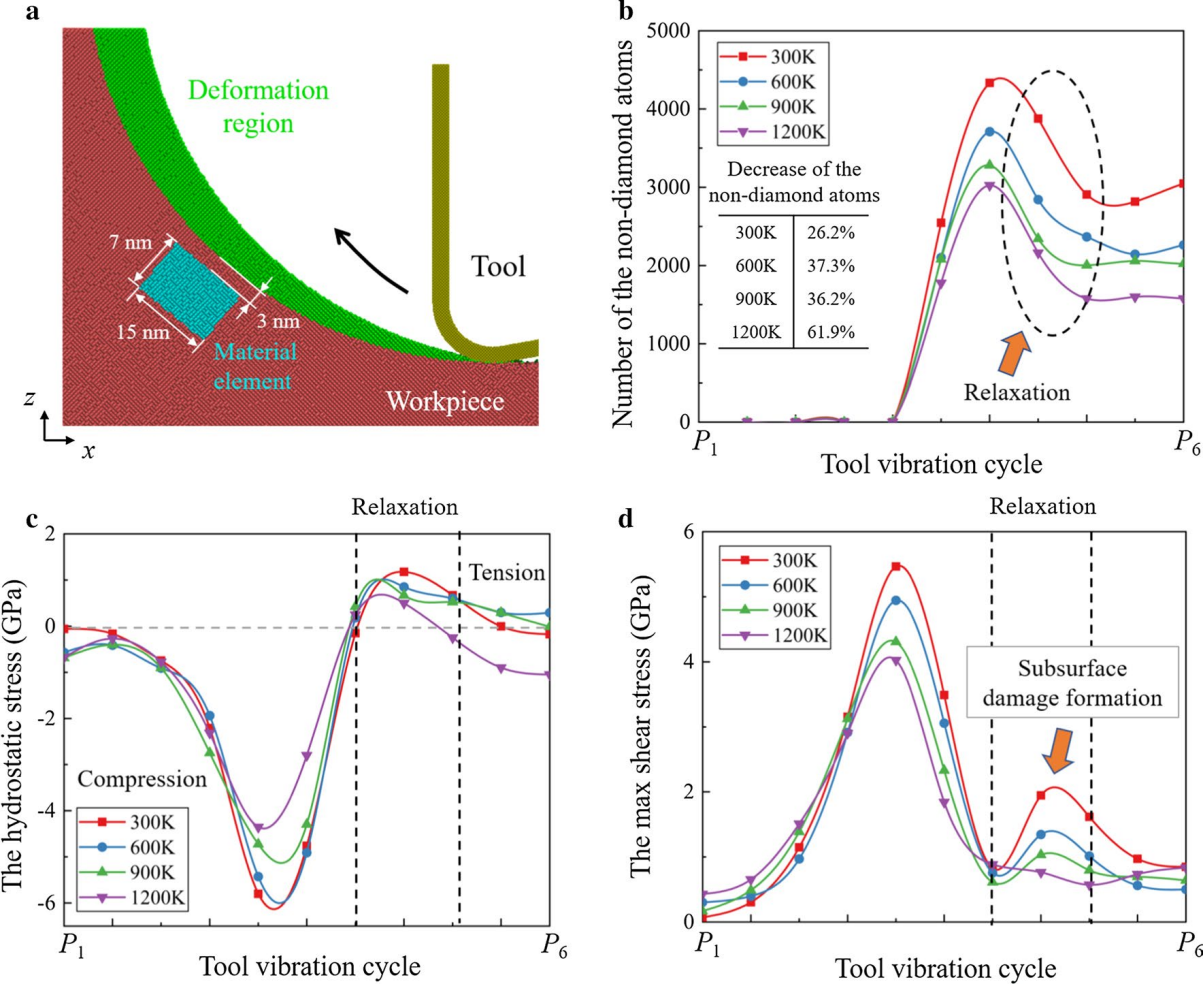
Relaxation during the HM process. **a** Illustration of the material element. **b** Statistics of the atoms in non-diamond structure in the material element. **c**, **d** Stress evolution of the material element

Further analysis of the vacancies is present in Fig. 10. A material element beneath the machined surface is chosen to monitor the vacancies generation. The number of atoms in non-diamond structure and stress evolution of the material element are present. It is concluded that during the cutting process, the material element is firstly compressed and then experiences tensile stress due to the tool upward movement. Meanwhile, two peaks of the shear stress can be observed at 300 K since the shear stress in the damage region is increased as cutting tool passed. When the cutting temperature is increased, the decrease of the shear stress is more obvious than tensile stress. At 1200 K, the second peak of the shear stress is nearly disappeared while tensile stress becomes dominant in the material element during the relaxation process.

**Fig. 11:**
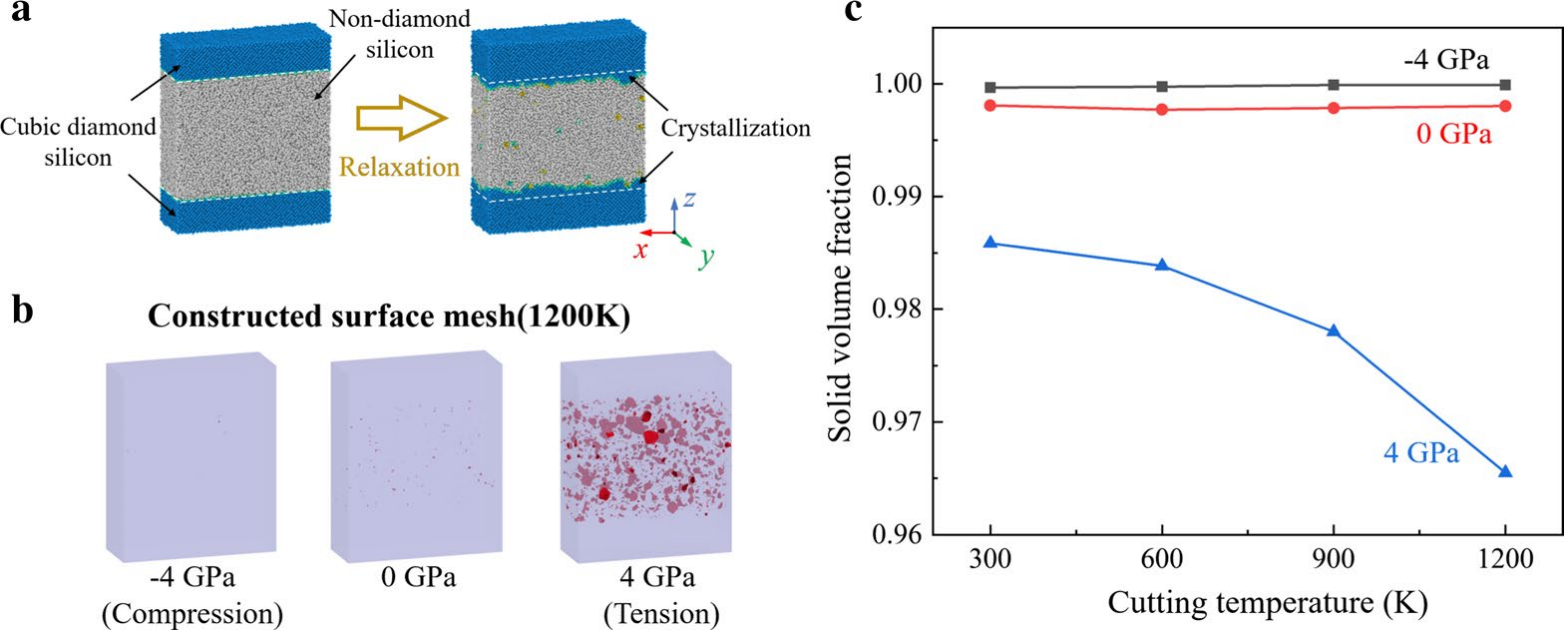
Relaxation simulation of silicon. **a** Scheme of MD relaxation simulation. **b** The surface mesh of the relaxed model at 1200 K. **c** The solid volume fraction curves

To explore the effect of tensile stress on formation process of the vacancies, relaxation simulations of bulk silicon sample were conducted. As shown in Fig. 11a, the initial model composes of 40% Si-I atoms and 60% a-Si atoms, which is generated by the melting-quench method [64]. The size of the model is 21.7 nm × 8.1 nm × 26.1 nm in *x*, *y*, and *z* direction, which contains 230,400 atoms. The initial interface between crystal and non-crystal region is set as (001) crystal plane. Periodic boundary condition is applied in three dimensions to mimic bulk materials. The constructed surface mesh of the relaxed model is present in Fig. 11b. Furthermore, to quantify the vacancies, the solid volume fraction is calculated as the ratio of the solid material volume and the total volume of the simulation sample, as shown in Fig. 11c. It is observed that when temperature is increased, the solid volume fraction decreased obviously under tensile stress. Therefore, to suppress the vacancies, the desired cutting temperature in HM process should be lower than that in ordinary TAC. Meanwhile, the vibration parameters should be optimized to reduce the tensile stress in subsurface workpiece.

## Conclusions

In this paper, MD simulation is carried out to investigate the cutting mechanism of hybrid machining (HM) process. A modified cutting model is applied to reveal the material removal behavior and subsurface damage formation in one vibration cycle. The main conclusions were as follows:

1. During HM process, the dominant material removal mechanism could transform from extrusion to shear in a single vibration cycle. With an increase of the cutting temperature, the generation and propagation of cracks can be effectively suppressed. However, the swelling appears when the dominant material removal mechanism becomes shearing, which is caused by the rotation of the crystal grains in workpiece.
2. Based on the stress analysis, the dominant formation mechanism of the subsurface damage in one vibration cycle can be distinct. In the extrusion stage, the subsurface damage can be generated by the shear stress in the damage region. While in the shearing stage, tensile stress becomes dominant in subsurface damage formation. When the cutting temperature is increased, although the tensile stress concentration in the subsurface workpiece is still obvious, the peak value of the stresses is apparently decreased, which effectively suppress the cracks and fractures in workpiece.
3. When the cutting temperature is increased, less subsurface damage is generated in the workpiece. However, due to the tensile stress, some vacancies can be generated in the workpiece when the cutting temperature is increased. Therefore, the desired cutting temperature during HM process should be lower than that in ordinary TAC and the vibration parameters should be set carefully to suppress the vacancies in the subsurface workpiece.

## Data Availability

The datasets used and analyzed in the current study can be obtained from the corresponding authors upon reasonable request.

## Abbreviations

TAC: Thermal assisted cutting
VAC: Vibration assisted cutting
LVC: Linear vibration cutting
EVC: Elliptical vibration cutting
DOC: Depth of cut
HM: Hybrid machining
FEM: Finite element method
MD: Molecular dynamics
LAMMPS: Large-scale Atomic/Molecular Massively Parallel Simulator
MEAM: Modified embedded-atom method
SW: Stillinger–Weber
COMB: Charge optimized many-body
ABOP: Analytical bond-order potential
Si-II: Metallic stable phase
HPPT: High-pressure phase transition
CNA: Common neighbor analysis
a-Si: Amorphous phase
Si-I: Single-crystal silicon phase
FCC: Face Center Cubic
CRSS: Critical resolved shear stress
